# Validation of a dynamic method of measuring households and populations from primary care Electronic Health Records: Cross-sectional comparison with Office for National Statistics Census 2021 estimates

**DOI:** 10.23889/ijpds.v8i2.2189

**Published:** 2023-09-14

**Authors:** Marta Wilk, Gill Harper, Nicola Firman, Chris Dibben, Rich Fry, Carol Dezateux

**Affiliations:** 1 Queen Mary University of London, London, United Kingdom; 2 The University of Edinburgh, Edinburgh, United Kingdom; 3 Swansea University, Swansea, United Kingdom

## Objectives

We have developed a dynamic method for identifying household members from Electronic Health Records (EHR). We compared the 2021 Census estimates of household number and demography with similar estimates derived from primary care EHRs on the Census date using primary care EHRs for the population of north east London (NEL).

## Method

We included 2,115,017 patients registered with a general practitioner on the 2021 Census date in NEL and assigned households from encrypted Unique Property Reference Numbers. We compared household number and size by Local Authority (LA), Middle Layer Super Output Area (MSOA) and area’s Index of Multiple Deprivation quintiles (IMDq) to Office for National Statistics (ONS) 2021 Census estimates and by LA to ONS Admin Based Housing Stock (ABHS) 2020 estimates. We assessed differences in EHR and Census 2021 populations by sex, age, LA, MSOA and IMDq. Sensitivity analyses will exclude those without a recent recorded clinical encounter.

## Results

EHR population estimates (2,115,017) were 116,346 (5.8%) higher than Census estimates (1,998,671), higher among men (9.2%) than women (2.5%) in almost all age groups, especially men aged 30-50 years and higher in the most (8.7%), than in the least (2.5%) deprived IMDq. EHR household estimates (660,789) were 68,047 (9.3%) lower than Census estimates (728,836), and 19,719 (3.1%) higher than ABHS occupied addresses (641,070). EHR household size estimates were 15.6%, 29.2%,12.5% and 8.4% lower for household sizes 1,2 3 and 4, and 13.3%, 42.1%, 82.1% and 195.8% higher for household sizes 5, 6,7 and 8 respectively when compared to Census estimates. EHR population and household estimates were respectively 5-10% higher and 5-11% lower for almost all NEL local authorities.

## Conclusion

EHR- and Census-derived population and household estimates differ, mainly in the prevalence of larger households. While data were extracted on the same date, person-level validation was not possible. Differences may reflect deregistration delay in EHR when changing residence. Analyses based on clinical encounters recency may identify registered patients who are no longer residents.

